# Case Report: C-reactive protein apheresis in non-ST-elevation ACS—case series from the C-reactive protein apheresis in Acute Myocardial Infarction Registry

**DOI:** 10.3389/fcvm.2024.1401566

**Published:** 2024-08-15

**Authors:** J. Torzewski, S. Mattecka, F. Esposito, F. Heigl, J. Fiedler, A. Sheriff

**Affiliations:** ^1^ Cardiovascular Center Oberallgaeu-Kempten, Clinic Association Allgaeu, Kempten, Germany; ^2^ Pentracor GmbH, Hennigsdorf, Germany; ^3^ Intensive Care and Cardiology, Gemeinschaftskrankenhaus Havelhöhe, Berlin, Germany; ^4^ Department of Apheresis, Medical Care Center Kempten-Allgäu, Kempten, Germany; ^5^ Gastroenterology/Infectiology/Rheumatology, Charité University Medicine Berlin, Berlin, Germany

**Keywords:** C-reactive protein apheresis, non-ST-elevation ACS, acute coronary syndrome, inflammation, anti-inflammatory therapy

## Abstract

C-reactive protein (CRP) apheresis has been introduced in ST-elevation myocardial infarction and cardiogenic shock. Here, we describe a first-in-man application in non-ST-elevation acute coronary syndrome (NSTE-ACS). Seven NSTE-ACS patients with high CRP levels (range 14.2–154 mg/L) were treated with CRP apheresis. Treatment was well-tolerated. Patients were discharged in good clinical condition.

## 1 Introduction

C-reactive protein (CRP) apheresis has recently been applied in ST-elevation myocardial infarction (STEMI) (1) and cardiogenic shock (CS) (2). The results of randomized controlled trials (RCTs) are eagerly awaited (3). In contrast to STEMI and CS, non-ST-elevation acute coronary syndrome (NSTE-ACS) has not yet been taken into consideration for such novel treatment. It is obvious, however, that similar results may be expected for NSTE-ACS as the underlying pathology, i.e., acute coronary stenosis/occlusion, is essentially the same (4). Thus, CRP apheresis may also preserve myocardial tissue in NSTE-ACS and may, in the long term, improve survival and quality of life.

Here, we describe seven consecutive NSTE-ACS patients treated within the C-reactive protein apheresis in Acute Myocardial Infarction Registry (CAMI-R) (https://drks.de/search/de/trial/DRKS00017481). As in CS and STEMI, CRP levels in NSTE-ACS patients rise significantly. This rise can be successfully counteracted using CRP apheresis.

## 2 Case description and diagnostic assessments

### 2.1 Study design

The CAMI-R has been extensively described elsewhere (2). Briefly, it is a prospective, multicenter, non-interventional trial (observational trial) to investigate the effect of CRP apheresis in patients with various subtypes of acute coronary syndromes and elevated CRP plasma levels (https://drks.de/search/de/trial/DRKS00017481). Thus, NSTE-ACS patients with elevated CRP plasma levels are also prospectively included. The patient's written informed consent is mandatory. Patients receive all treatment in accordance with the AHA/ESC guidelines (4). The follow-up includes medical visits at patient discharge and phone calls after 30 days and 1 year.

### 2.2 NSTE-ACS diagnosis

NSTE-ACS according to AHA/ESC guidelines was suspected in patients with ACS without ST elevations on ECG (4). A suspected diagnosis was confirmed using coronary angiography demonstrating coronary stenosis and impaired blood flow. Patients were treated with percutaneous coronary intervention (PCI) according to AHA/ESC guidelines (4).

### 2.3 C-reactive protein apheresis

CRP apheresis has been extensively described elsewhere (1–3, 5). It was performed with a selective CRP adsorber (PentraSorb® CRP; Pentracor GmbH, Hennigsdorf, Germany). Briefly, patients received two to four sessions of CRP apheresis after PCI and admission to the Chest Pain Unit (CPU). The first apheresis started 62.1 ± 35.3 h after onset of symptoms (OoS), and the second apheresis was approximately 16–20 h later. A third or fourth apheresis was performed if, in another 12 h later, the CRP plasma concentration rose to values above 30 mg/L. This protocol largely followed the CAMI-1 protocol for STEMI patients (1). Again, approximately 6,500 ml of plasma was processed in each apheresis session, preferentially in cycles of 500 ml between the regeneration of the CRP adsorber.

### 2.4 C-reactive protein quantification

All participating diagnostic laboratories were accredited by German/European law (1–3, 5), and CRP was quantified with standard procedures. The CRP tests remained the same throughout the study. The concentration of CRP plasma was adjusted using the hematocrit of the same blood sample.

### 2.5 In-hospital mortality and 30-day follow-up

In-hospital mortality was assessed by medical visit at patient discharge and the 30-day follow-up was carried out by telephone (2).

## 3 Results

### 3.1 Patients

Seven patients (mean age 57.1 ± 7.3 years) with the diagnosis of NSTE-ACS and a significant rise in CRP plasma levels were included into the CAMI-R (Table 1). Previous PCI was reported in one patient. All patients were classified as Killip Class I. Out of seven patients, four received ramus circumflexus (RCX)-PCI. The time between OoS and PCI varied considerably (mean 31.5 ± 35.1 h). This is explained by the fact that RCX occlusion tends to be invisible on 12-channel ECG, and clinical presentation and cardiac enzymes only reveal the final NSTE-ACS diagnosis resulting in interventional treatment (4). Nonetheless, pathophysiology is analogous to STEMI in other regions of cardiac blood supply [ramus interventricularis anterior (RIVA)/left anterior descending artery (LAD) or right coronary artery (RCA)] (4).

**Table 1:** Patient characteristics.

Characteristics	(%)
Age (years)	57.1 ± 7.3
Sex (male/female)	6 (85.7)/1 (14.3)
Cardiovascular risk factors	
Smoker (yes/formerly)	5 (71.4)/2 (28.6)
Diabetes	1 (14.3)
Hypertension	4 (57.1)
Dyslipidemia	3 (42.9)
Former PCI	1 (14.3)
Acute PCI	
LAD/RIVA	2 (28.6)
RCX (circumflex)	4 (57.1)
RCA (right)	1 (14.3)
Multi-vessel disease (>50%)	4 (57.1)
Second PCI (within 30 days)	1 (14.3)
GRACE scores	78.43 ± 18.5
Troponin-T peak (pg/ml)	1,735.87 ± 1,380.99
LVEF at discharge (>50%)	6 (85.7)
LVEF at discharge (40%–50%)	1 (14.3)
Mortality (in-hospital, 30 days)	0 (0)
Killip Class (all patients)	I
OoS to stent (h)	31.5 ± 35.1
Admission to discharge (d)	5 ± 2.6
CRP 24 h after OoS (mg/L)	14.2 (2.4–122.2)
30 days follow-up (including death, stroke, unstable angina, re-admission/heart failure, bypass surgery, pacemaker/ICD implantation)	0 (0)
P2Y12 inhibitors	
Clopidogrel (due to parallel edoxaban treatment)	1 (14.3)
Prasugrel	2 (28.6)
Ticagrelor	4 (57.1)

LVEF, left ventricular ejection fraction; ICD, implantable cardioverter defibrillator.

### 3.2 C-reactive protein apheresis

**Figure 1:**
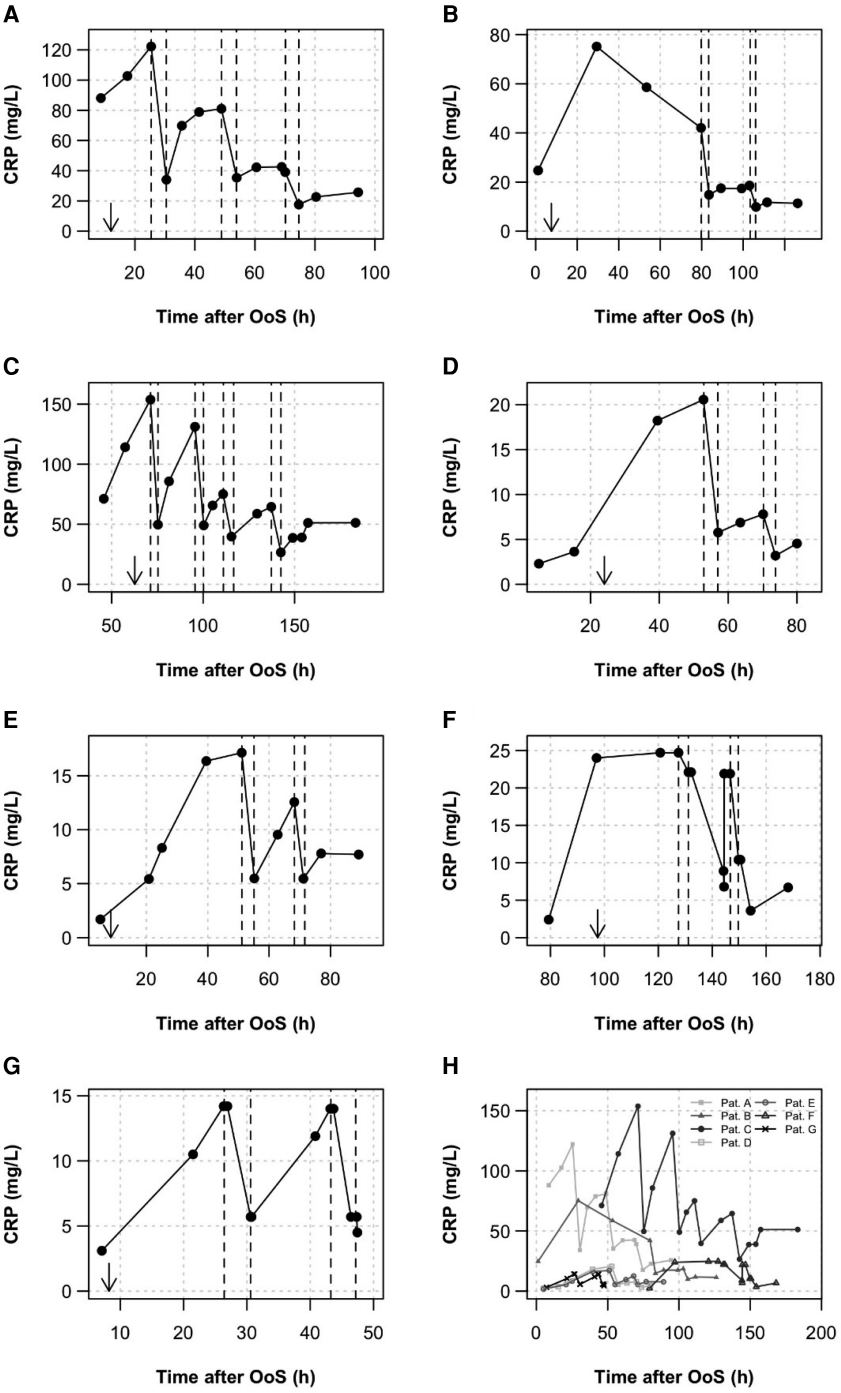
CRP kinetics. CRP was measured routinely during the clinical stay. (**A**–**G**) All kinetics are depicted. Apheresis treatments are shown as dashed lines. Arrows indicate the time of PCI. Patients received two to four CRP apheresis sessions after PCI and admission to the CPU. The first apheresis started at a mean of 62.1 ± 35.3 h after OoS, the second apheresis was approximately 16–20 h later. A third or fourth apheresis was performed if, another 12 h later, the CRP plasma concentration rose to values above 30 mg/L. (**H**) Summary diagram showing high variability of CRP levels in NSTE-ACS.

C-reactive protein levels in seven consecutive NSTE-ACS patients are shown in Figure 1. If plasma levels rose above 10 mg/L, CRP apheresis and inclusion into the CAMI-R were recommended to the patient. As depicted in Figure 1, CRP plasma levels after NSTE-ACS were highly variable, in the range of 13–150 mg/L. The highest levels were observed in patients A and C. Both patients experienced RCX (circumflex) stenosis/occlusion. However, patient C had concomitant infection with severe acute respiratory syndrome coronavirus 2 (SARS-CoV-2) (6, 7). The mean CRP concentration 24 h after OoS was 42.4 ± 47.1 mg/L (Table 1). CRP apheresis was performed two to four times until the CRP levels reached a plateau and did not rise significantly again.

All apheresis parameters are summarized in detail in Table 2. The seven patients received a total of 17 apheresis treatments, in the range of two to four sessions per patient. Apheresis was well tolerated with no relevant side effects. The first apheresis treatment started at 62.1 ± 35.3 h after OoS. Further apheresis sessions were performed at time intervals of approximately 16–20 h. Treatments lasted 3.9 ± 0.8 h. The relative plasma volume processed was 1.7 ± 0.5 per apheresis. CRP depletion was 57.9% ± 8.3% in total. After the final session of apheresis, a CRP concentration of <20 mg/L was achieved in six patients, and in one patient treatment finalized at a CRP concentration of <27 and >20 mg/L.

**Table 2:** Apheresis parameters.

	First apheresis (*n* = 7)	Second apheresis (*n* = 7)	Third apheresis (*n* = 2)	Fourth apheresis (*n* = 1)	All (*n* = 17)
Time between OoS and start of apheresis (h)	62.1 ± 35.3	82.3 ± 35.9	90.6 ± 29.0	137.4	—
Time interval between aphereses (h)	—	16.2 ± 3.3	13.5 ± 3.9	20.6	—
Duration of treatment (h)	4.1 ± 0.5	3.7 ± 0.8	5.1 ± 0.8	5.2	3.9 ± 0.8
Total plasma volume processed (L)	6.5 ± 0.9	5.8 ± 1.3	7.5 ± 0.7	8.0	6.4 ± 1.9
Relative plasma volume processed	1.8 ± 0.1	1.6 ± 0.3	2.0 ± 0.2	2.1	1.7 ± 0.5
CRP before apheresis (mg/L), median (range)	24.7 (14.2–154)	18.6 (7.8–131)	57.1 (39.1–75.1)	64.5	24.7 (7.8–154)
CRP depletion (%)	**62.6 **±** 10.1**	**55.3 **±** 5.0**	**50.1 **±** 4.5**	**58**.**2**	**57.9 ± 8.3**

Values are given as mean ± SD or median with range. Depletion is normalized on hematocrit.

Bold values indicate % CRP depletion during apheresis session.

### 3.3 Endpoints

#### 3.3.1 In-hospital mortality

Patients were discharged within a mean of 5 ± 2.6 days. No in-hospital mortality was observed.

#### 3.3.2 The 30-day follow-up

The telephone follow-ups revealed no 30-day mortality.

## 4 Discussion

CRP apheresis is now a promising novel intervention to protect hypoxic but still living, recoverable cells from CRP-triggered opsonization for Fcγ-R mediated phagocytosis by macrophages (1–3, 5–8). CRP may thus serve as a primitive antibody that binds to lysophosphatidylcholine expressed on the surface of hypoxic/apoptotic cells (3, 8). The latter pathomechanism may be relevant in various human diseases but is still best studied in STEMI and CS (1, 2). Although the pathomechanism is analogous in most cases, NSTE-ACS has not yet been considered for treatment with CRP apheresis (4).

Here, we describe a first-in-man application of CRP apheresis in NSTE-ACS within the CAMI-R. We do focus on the feasibility and safety of the treatment. Importantly, treatment success can only be proven in an RCT (1–3).

Some features in this case series are intriguing:

First, CRP apheresis in NSTE-ACS is well tolerated, and patients were discharged in good clinical condition.

Second, CRP apheresis is feasible and safe, in case of concomitant viral or bacterial infection (6, 7). In our case series, no correlation with leukocyte count was observed (please see the Supplementary Material). COVID-19 (Coronavirus 2019), diagnosed in patient C, causes a rise in CRP levels. SARS-CoV-2 with acute respiratory distress syndrome (ARDS) during the early phase of the recent pandemic caused a surprisingly high rise of CRP levels indeed (6, 7). The latter was unusual for a viral disease. Later, with virus attenuation caused by the selection of less virulent mutants, CRP levels in patients were much lower. Patient C experienced a mild SARS infection with only flu-like symptoms. Importantly, high CRP levels in the context of myocardial infarction may increase myocardial damage independent from the cause of the CRP rise. The latter should be considered in the design of future clinical trials.

Third, four out of seven patients had RCX (circumflex) stenosis/occlusion with markedly elevated CRP plasma levels. Clearly, the underlying pathology in STEMI and NSTE-ACS is often analogous, and ECG is the only formal criteria of discrimination (4). CRP levels in NSTE-ACS patients vary a lot more than CRP levels in STEMI patients. This may be due to the varying extent of myocardial damage (caused by complete or only partial coronary occlusion) and also to the varying time points of coronary intervention following AHA/ESC guidelines. As complete coronary occlusion is more common in STEMI, CRP levels after STEMI may be higher in general. The latter requires further analysis.

Alternative attempts to inhibit CRP remain difficult (9). Thus, CRP apheresis should be considered a potential adjunct to the therapeutic armamentarium of NSTE-ACS treatment in future clinical trials.

## 5 Patient perspectives

1. Novel aspects of inflammation/CRP in NSTE-ACS.
2. Potential therapeutic role of CRP apheresis in NSTE-ACS.

## Data Availability

The raw data supporting the conclusions of this article will be made available by the authors, without undue reservation.
