# Supplementary figures and images for: Case Report: C-reactive protein apheresis in non-ST-elevation ACS—case series from the C-reactive protein apheresis in Acute Myocardial Infarction Registry
Source: Front Cardiovasc Med. 2024 Aug 15;11:1401566. doi: 10.3389/fcvm.2024.1401566 (PMC11357922; doi:10.3389/fcvm.2024.1401566)

CamiReg-01-040

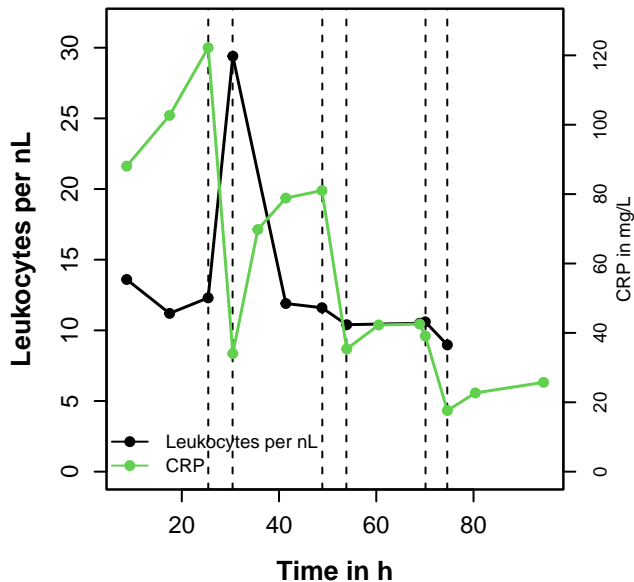

CamiReg-01-043

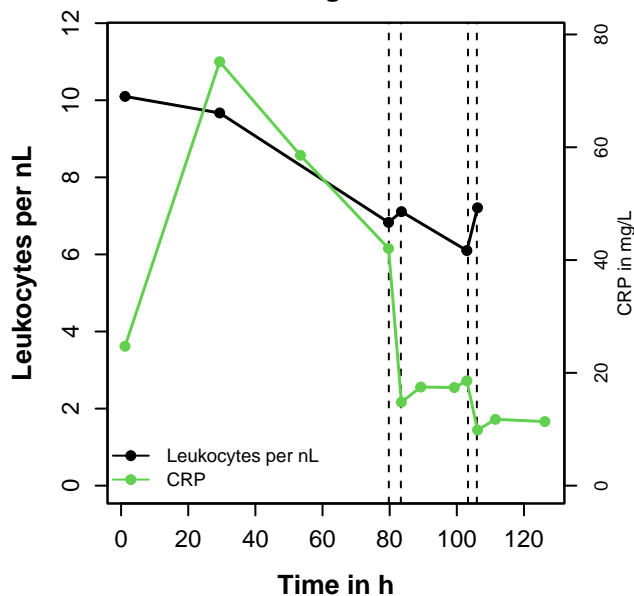

CamiReg-01-045

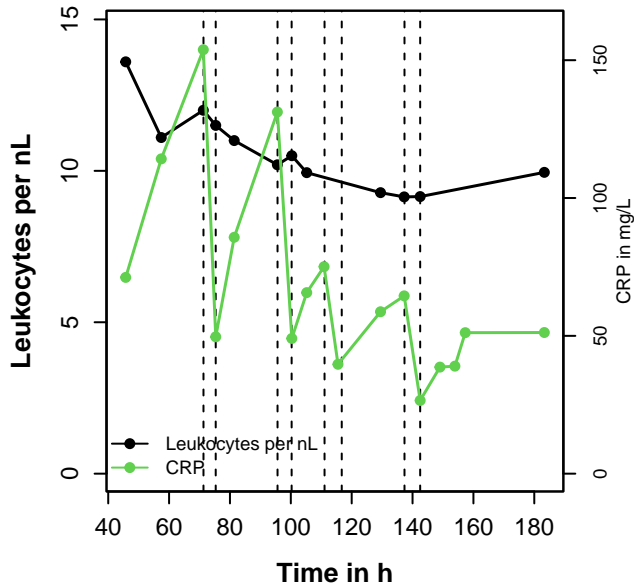

CamiReg-01-046

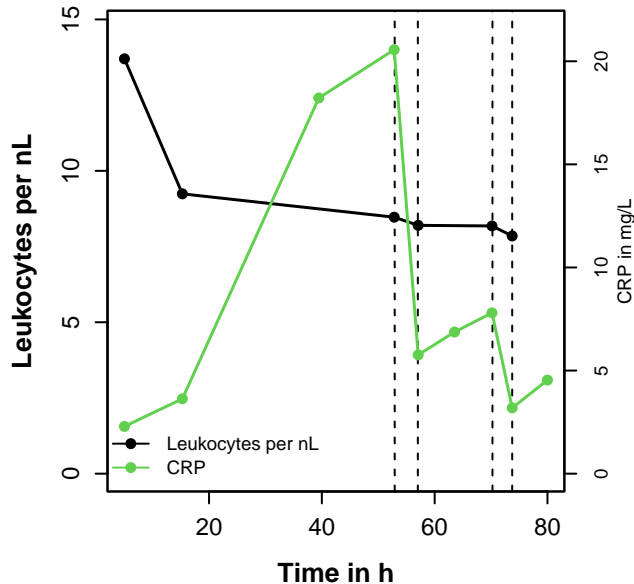

CamiReg-01-048

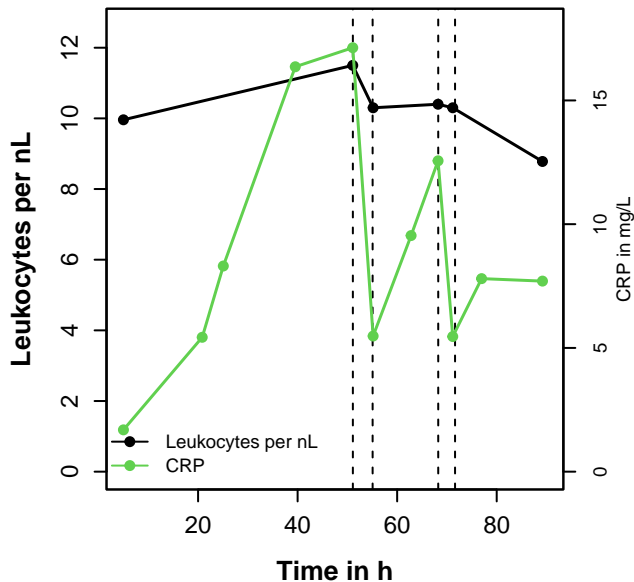

CamiReg-10-001

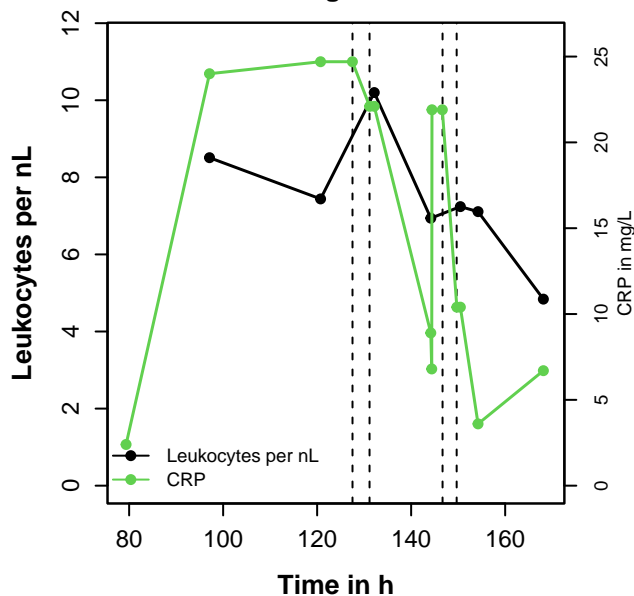

CamiReg-10-007

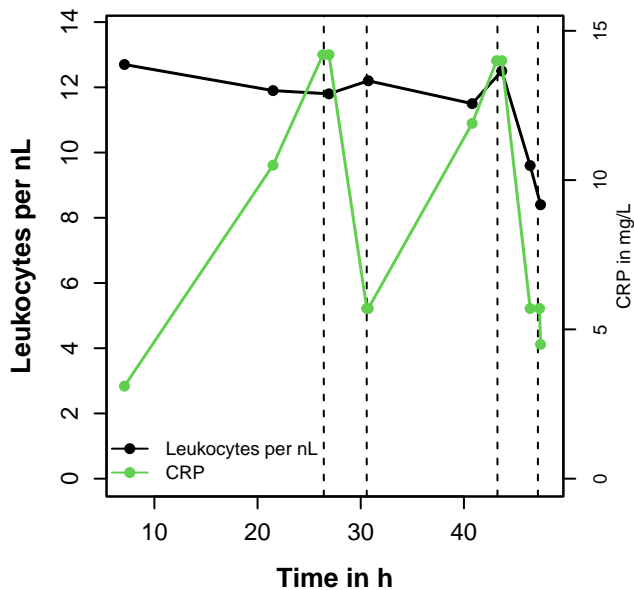

Supplement: Supplementary file 1 [file Datasheet1.pdf]
